# Retraction of “Down-regulation of miR-539 indicates poor prognosis in patients with pancreatic cancer”

**DOI:** 10.1515/biol-2025-0004

**Published:** 2025-11-25

**Authors:** Haibo Yu, Hongliang Song, Zhongwu Ma, Wu Ji

**Affiliations:** Research Institute of General Surgery, Nanjing General Hospital of Nanjing Military Region, the First School of Clinical Medicine, Southern Medical University, Nanjing, 210002, PR China; Department of Hepatobiliary Surgery, Wenzhou Central Hospital, The Dingli Clinical Institute of Wenzhou Medical University, Wenzhou, 325000, PR China

Retraction of: Yu H, Song H, Ma Z, Ji W. Down-regulation of miR-539 indicates poor prognosis in patients with pancreatic cancer. Open Life Sciences 2018; 13 (1): 497–503, DOI: 10.1515/biol-2018-0059.

This article has been retracted by agreement between the Editor-in-Chief of Open Life Sciences, Thomas Litman, and the publisher, Walter De Gruyter GmbH, following an editorial investigation conducted in line with the Committee on Publication Ethics (COPE) Retraction Guidelines.

The retraction follows identification of ethical flaws in the study’s scientific design. The article reports surgical resections carried out in patients later confirmed as having stage IV pancreatic cancer. International guidelines, including the WMA Declaration of Helsinki, specify that resection is not appropriate once distant metastases are present. Reliance on data derived from procedures that diverge from these standards raises serious concerns about the ethical and clinical validity of the work.

In addition, discrepancies were observed between the published results and the supporting documentation, collectively resulting in a loss of confidence in the integrity of the presented data.

